# An Evil Which Must Be Ended: A Colonial Experience

**Published:** 1919-01-25

**Authors:** 


### An Evil Which Must be Ended: A Colonial Experience.

*To the Editor of* THE HOSPITAL.

SIR,—As an instance of the extreme to which day nursing in the night can be carried I offer an experience of my own. As the result of an accident I was in a Colonial hospital for surgical operation, and by way of privilege accorded to me as a nurse-matron was given a private ward for which to ordinary patients a charge of some seven guineas or more weekly was made. It was a large hospital training-school and the private ward department was a special feature. To two wards of one patient each there was one certificated nurse by day and one probationer by night. Yet both patients were washed and "done" generally before 6 A.M.—by the probationer—who gave an hour to each, one taking up from four to five o'clock, the other from five to six. This hour was given on principle —whether really needed or not—by way to teaching the probationer the extra details of attention which the private patient might expect.

Once the second patient in my nurse's charge—my next-door neighbour, that is—was a man, whose operation was of a most painful nature. For two whole days and nights his cries of pain were unceasing, distressing all within hearing, which was the whole floor of private patients. All the morphia that dared be given him gave him no relief. Then on the third night toward 2 A.M. there was blessed silence. I lay awake enjoying this when the night nurse came to me, and we agreed upon the blessedness it was, not only to have peace ourselves, but to know that the sufferer at length had found it. I fell asleep and was duly awakened at four. At five as the nurse finished me she remarked how sorry she was now to have to disturb the man. I said, "Surely you *won't* disturb him?" Her reply was, "I shall have to. I go off at six, he *must* be done by then. And, anyway, there's one comfort—*he's had three hours' sleep.*" The man was wakened, and his cries lasted many hours again, until morphia once more relieved him.

